# Presidential address: recent advance of mycorrhizal research in China

**DOI:** 10.1080/21501203.2018.1437838

**Published:** 2018-02-09

**Authors:** Liang-Dong Guo

**Affiliations:** a State Key Laboratory of Mycology, Institute of Microbiology, Chinese Academy of Sciences, Beijing, China; b Life Sciences of College, University of Chinese Academy of Sciences, Beijing, China

**Keywords:** Presidential address, mycorrhizal fungi, recent progress

## Abstract

I am honoured to address as the seventh president of the Mycological Society of China. Mycorrhizal research has a long history in China, including taxonomy, diversity, ecology, molecular biology, and application. Particularly in the past four decades, great progress in mycorrhizal field has been made by Chinese mycologists and ecologists. In this paper, through my own experience, I summarised the main and important advance of recent mycorrhizal researches in terms of mycorrhizal fungal diversity, community, responses to global environmental changes, molecular biology, and function in China. Some perspectives are also proposed for future mycorrhizal studies in China.

## Mycorrhizae

Mycorrhizae are symbiotic structures formed between soil fungi and terrestrial plant roots (Smith and Read 2008). In these associations, the plants provide carbon and lipids for the growth and function of fungi (Bago et al. 2000; Jiang et al. 2017), and thus can influence the fungal community via host specificity, producing diverse organic substrates and modifying microhabitats. In return, mycorrhizal fungi improve plant nutrient (particularly in nitrogen and phosphorus) and water uptake and resistance to biotic and abiotic stresses, and they therefore influence plant diversity, productivity, and ecosystem functioning by forming underground common mycorrhizal networks that connected individuals of plants.

**Figure 1.**
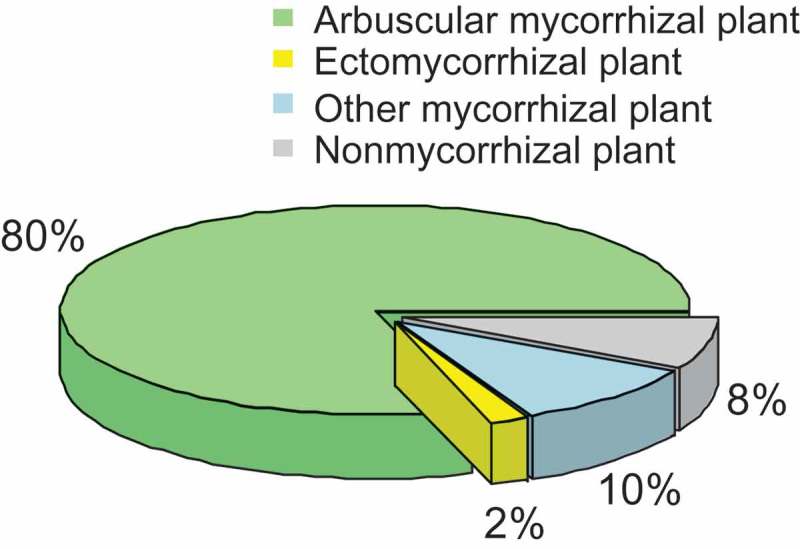
The proportion of different types of mycorrhizal plant species.

According to dissection characteristics and host plants, mycorrhizae are divided into seven types, i.e. arbuscular mycorrhiza (AM), ectomycorrhiza (EM), ectendomycorrhiza, orchid mycorrhiza, arbutoid mycorrhiza, monotropoid mycorrhiza, and ericoid mycorrhiza (Smith and Read 2008). Based on review of previous studies, it estimates that ca. 50,000 fungal species form mycorrhizal associations with ca. 250,000 plant species in the world. For example, there are ca. 300 described AM fungal species (Glomeromycota) based on morphological characteristics of the spores, and up to 1600 AM fungal operational taxonomic units based on environmental rDNA sequences by using molecular techniques. These AM fungal taxa form symbiotic associations with ca. 80% of plant species (Figure 1). It is estimated that there are ca. 20,000 EM fungal species, mainly belonging to Basidiomycota, followed by Ascomycota and Zygomycota, according to morphological and molecular techniques, which form symbiotic associations with ca. 2% of plant species, such as Pinaceace, Fagaceae, Betulaceae, Salicaceae, and Dipterocarpaceae (Figure 1). The other mycorrhizal types occupy ca. 10% of plant species (Figure 1). There is ca. 8% of plant species belonging to non-mycorrhizal plants, such as Brassicaceae, Crassulaceae, Haemodoraceae, Orobanchaceae, Proteaceae, and Restionaceae (Figure 1).

## Mycorrhizal research progress in china

**Figure 2.**
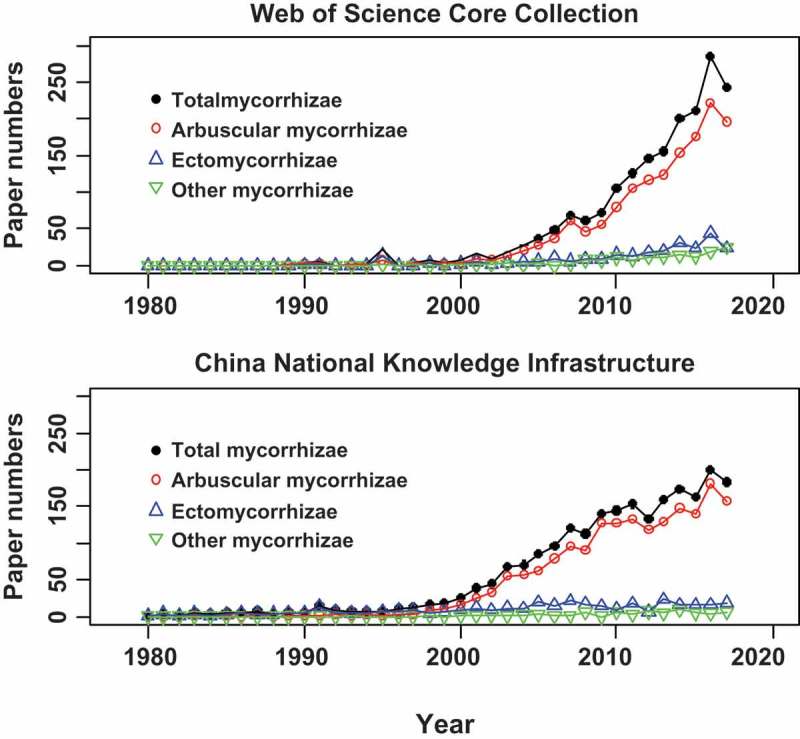
The publication on mycorrhizae by Chinese researchers during years 1980−2017.

There is a long history of mycorrhizal research in China, including taxonomy, diversity, ecology, molecular biology, and application. For example, a total of 1893 science citation index (SCI) papers (Web of Science Core Collection) and 2272 papers in Chinese Journals (China National Knowledge Infrastructure, CNKI) have been published by Chinese scientists since 1980 (searched on 6th December, 2017) (Figure 2). Particularly, the published papers have greatly increased since 2000, indicating that progress in mycorrhizal research was apparently boomed recently. In 2017, two special issues focusing on “mycorrhizal fungi” and “mycorrhizal fungi and plant resistance” were published in Chinese mycological journals of “Mycosystema” and “Journal of Fungal Research”, respectively, showing a portion of advances in taxonomy, species diversity, ecology, physiology, and functioning of mycorrhizal fungi were made by Chinese researchers. The recent progress of mycorrhizal research in China was summarised in this paper.

## Mycorrhizal fungal diversity

A total of ca. 147 AM fungal species and ca. 500 EM fungal species were described based on morphological characteristics in China (He et al. 2010a; Wang and Liu 2017). Although many studies have been carried out in the identification of mycorrhizal fungi based on morphological characteristics, higher species diversity of mycorrhizal fungi was found from soils and plant roots in various ecosystems using molecular techniques. For example, a total of 66 and 26 EM fungal taxa were found from roots of *Quercus liaotungensis* and *Pinus tabulaeformis* in a temperate forest ecosystem using direct internal transcribed spacer (ITS) sequencing of EM root tips (Wang and Guo 2010; Wang et al. 2012). By using 454 pyrosequencing, an average of 37.8 AM fungi was obtained from each of 17 woody plant species (Chen et al. 2017), and an average of 32.8 EM fungi was found in each of 12 mixed root samples (Gao et al. 2015) in a subtropical forest. In the agro-ecosystems, there were average 10−16 and 22−27 AM fungi observed in maize or rice root and soil samples, by using terminal restriction fragment length polymorphism and/or clone library analyses (Liu et al. 2014, 2016; Wang et al. 2015), but an average 29−49 AM fungi was detected in soil samples by using 454 pyrosequencing technique (Lin et al. 2012). In the grassland ecosystems, a total of 34−37 AM fungi was obtained from mixed plant root samples in alpine meadow ecosystems via clone library method (Liu et al. 2012b; Yang et al. 2013). However, higher AM fungal richness of 48−54 and 72−79 were subsequently detected in soil samples collected from the alpine meadow (Zheng et al. 2014, 2016b) and semiarid steppe (Kim et al. 2015; Li et al. 2015), using 454 pyrosequencing.

In maintenance mechanism of mycorrhizal fungal diversity, Gao et al. (2013) investigated EM fungal diversity in the plot of different diversity levels of host plants and found host genus-level diversity could be the best predictor of EM fungal diversity in a subtropical forest. Further study in this subtropical forest ecosystem showed that EM fungal diversity was significantly affected by the basal area of the dominant host plant species *Castanopsis eyrei* in the ridge habitat, but by the basal area of total host plant species in the valley habitat, and this study suggests that the relationship of EM fungal diversity and plants is dependent on habitat types (Gao et al. 2017). In addition, Wang et al. (2015) reported that the AM fungal diversity in the roots of rice increased with the growth of rice, due to progressive colonisation and aerenchyma development of rice in paddy wetland ecosystem.

## Mycorrhizal fungal community

In the dynamics and assembly mechanism of mycorrhizal community, Wang et al. (2012) found that EM fungal community composition of *Q. liaotungensis* was not significantly different in different forest ages and seasons in a temperate forest ecosystem. The EM fungal community composition was significantly different in young forest (<40 years) from intermedium (41−80 years) and old (>80 years) forests, and the EM fungal community assembly was significantly structured by environmental selection in the young and intermedium forests, but by environmental selection and dispersal limitation in the old forest in a subtropical ecosystem (Gao et al. 2015). Further study in this subtropical forest ecosystem showed that EM fungal composition was significantly affected by plant species composition and geographic distance in the ridge habitat but by soil pH in the valley habitat; in contrast, AM fungal community composition was not significantly influenced by any variables investigated in the ridge and valley habitats (Gao et al. 2017). In addition, ecological network study demonstrated that woody plant-AM fungi mutualistic network was highly interconnected and nested but in anti-modular and anti-specialised manners, and the non-random pattern was explained by plant and AM fungal phylogenies, with a tendency for a stronger phylogenetic signal by plant than AM fungal phylogeny in the subtropical forest ecosystem (Chen et al. 2017).

## Mycorrhizal fungal response to global change

Global change affects plant species diversity, production and community and ecosystem function. Similarly, global change can influence mycorrhizal fungi in ecosystems. For example, warming had neutral, negative or positive effects on AM fungal root colonisation rate, spore density, extra-radical hyphal (ERH) density, and richness (Yang et al. 2013, 2016; Kim et al. 2014, 2015; Gao et al. 2016; Shi et al. 2017a), and changed AM fungal community composition in soil rather than in root (Yang et al. 2013) in grassland ecosystems. Fertilisation (nitrogen, phosphorus, or organic manure) significantly decreased AM fungal root colonisation rate, spore density and ERH diversity, and altered AM fungal community composition in agricultural (Lin et al. 2012) and grassland (Liu et al. 2012b; Zheng et al. 2014; Kim et al. 2015) ecosystems. Increased precipitation could significantly increase AM fungal root colonisation rate, spore density, and ERH density, decrease AM fungal diversity, and changed AM fungal community composition in grassland ecosystems (Li et al. 2015; Gao et al. 2016). Land use conversion from grassland to farmland significantly reduced soil AM fungal ERH density and richness, and altered AM fungal community composition (Xiang et al. 2014). In addition, grass (*Elymus nutans, Avena sativa*, and *Vicia sativa*) plantation affected rhizosphere soil AM fungal ERH density, richness and community composition (Zheng et al. 2016b).

## Molecular biology of mycorrhizal fungi

In the mycorrhizal molecular biology, Li et al. (2013) firstly cloned and identified two aquaporin genes of *GintAQPF1* and *GintAQPF2* from AM fungus *Glomus intraradices* (syn. *Rhizophagus intraradices*) and confirmed their function under drought stress. Liu et al. (2015) found that drought stress upregulated the level of *R. intraradices* and soybean mitogen-activated protein kinase (MAPK) transcripts in mycorrhizal soybean roots, indicating the possibility of a molecular dialogue between AM fungus and host plant and suggesting that they might cooperate to improve soybean’s resistance to drought stress. Concurrently, in terms of nutrient absorption, some studies investigated the mycorrhiza-regulated phosphate transporters in host plants *Astragalus sinicus* (Xie et al. 2013), *Oryza sativa* (Sun et al. 2012), *P. tabulaeformis* (Zheng et al. 2016a), pepper and tobacco (Chen et al. 2007), and reciprocal transportation of carbon and phosphorus amongst plants (*Zea mays* and *Medicago sativa*), *Rhizophagus irregularis* and phosphate-solubilising bacterium (Zhang et al. 2016; Wang et al. 2016a). Wang et al. (2014) demonstrated that H^+^-ATPase played a key role in energising the periarbuscular membrane, thereby facilitating nutrient exchange in arbusculated rice and *Medicago truncatula* cells. Interestingly, GigmPT from *Gigaspora margarita* was expressed in the arbuscules and intraradical hyphae, and silencing of this gene inhibited the arbuscule formation, suggesting that GigmPT was involved in phosphate (Pi) reuptake and was required for AM symbiosis (Xie et al. 2016). From plant *Lycium barbarum*, a drought-tolerant, perennial ligneous shrub, Hu et al. (2017) characterised three proteins (LbPT3, LbPT4, and LbPT5) which were involved in the mycorrhizal Pi pathway and could be inhibited by Pi supply but not regulated by water stress. Jiang et al. (2017) showed that AM fungus *R. irregularis* was a fatty acid auxotroph and that fatty acids synthesised in plant (*M. truncatula*) were transferred to the fungus. Moreover, Chinese scientists have made significant progress in terms of mycorrhizal signal transduction, for example, some specific proteins such as DELLA and CERK1, were found to be common components of symbiotic mycorrhizal signalling pathways and to play critical function during the establishment of symbiosis between AM fungi and host plants (e.g. Yu et al. 2014; Zhang et al. 2015; Jin et al. 2016).

## Mycorrhizal function

Mycorrhizal fungi play an important role in promoting plant growth and resistance to abiotic stresses. For example, AM fungi can efficiently improve crop growth, biomass, and productivity particularly in extremely P-limited soil (Hu et al. 2009), due to enhanced nutritional assimilation of plant as well as improved soil organic matter content and total nitrogen (Wu et al. 2005). AM fungi can enhance plant resistance to drought (Li et al. 2013; Liu et al. 2015) and heavy metals (Dong et al. 2008; Zhang et al. 2009). AM fungus *Glomus mosseae* (syn. *Funneliformis mosseae*) could alleviate the side effects induced by fungicide chlorothalonil on upland rice growth (Zhang et al. 2006). Moreover, AM fungus *R. intraradices* (formly *G. intraradices*) was found to significantly decrease the concentration of organophosphate insecticide named phoxim in shoots and roots of carrot and green onion (Wang et al. 2011). Liu et al. (2012a) demonstrated that dual inoculation of specific AM fungi and plant growth-promoting rhizobacteria (PGPR) could stimulate each other and that the combination of *G. mosseae* (syn. *F. mosseae*) and PGPR *Bacillus* sp. could interact to maximally suppress the root-knot nematode (i.e. *Meloidogyne incognita*) and tomato disease development. Recently, He et al. (2017) reported that AM fungi and their indirect interactions with insect (*Spodoptera exigua*) altered the photosynthesis and plant endogenous hormones of peanut and tomato. Additionally, the application of mycorrhizal inoculation was found to improve carbon sequestration of coalfield soils and thus to be beneficial for the ecological reclamation in mine region (Wang et al. 2016b). The glomalin-related soil protein produced by AM fungi is not only an important carbon sink, but also may it be useful indicators for evaluating soil quality and function of desert ecosystem (He et al. 2010b).

Mycorrhizal fungi play important roles in interspecific competition in a subtropical forest ecosystem. For example, AM fungi (i.e. *Diversispora eburnea, Claroideoglomus lamellosum, F. mosseae*, and *Diversispora* sp.) significantly promoted a competitive advantage of *Rhus chinensis* over both *Celtis sinensis* and *Cinnamomum camphora* (Shi et al. 2016). EM fungi (*Paxillus involutus, Pisolithus tinctorius, Cenococcum geophilum*, and *Laccaria bicolor*) significantly promoted a competitive ability of the mid-successional tree *Cyclobalanopsis glauca* over the pioneer tree *Pinus massoniana* compared with the uninoculated control treatment (Shi et al. 2017b). These findings suggest that the extent to which mycorrhizal fungi affected interspecific plant competition outcomes was dependent on mycorrhizal fungus identity.

In summary, despite many innovative studies and great progresses in mycorrhizal field have been made in the past several decades in China, we still need to make efforts, at least, in the following aspects: (1) to conduct more wide and deep researches on the mechanism of mycorrhizal fungal diversity maintenance and community assembly, (2) to strengthen the studies on molecular interaction mechanisms between mycorrhizal fungi, host plants, and other organisms, (3) to improve understanding of mycorrhizal fungal response and adaptive mechanisms under global environmental changes, especially to uncover possible alterations of ecological functioning under interactive conditions of multiple global change factors, and (4) to increase the application of mycorrhizae. I believe that, with increasing of joined Chinese young mycorrhizal scientists and financial support by Chinese government, Chinese scientists will make great progress in mycorrhizal field in future.
